# Supplementary material for: Stigma and its impact on disclosure and mental health secrecy in young people with clinical depression symptoms: A qualitative analysis
Source: PLoS One. 2024 Jan 5;19(1):e0296221. doi: 10.1371/journal.pone.0296221 (PMC10769096; doi:10.1371/journal.pone.0296221)
Supplement: Attachment — Submitted filename: Response to Reviewers.pdf [file pone.0296221.s001.pdf]

UNIVERSITY OF READING
SCHOOL OF PSYCHOLOGY AND CLINICAL LANGUAGE SCIENCES

Katie Prizeman
Schwerzenbachstrasse 21
8117 Fällanden
Zürich, Switzerland
Research Student: Doctor of Philosophy in Psychology
School of Psychology and Clinical Language Sciences
TEL: +41 76 304 0711
EMAIL: k.c.diab@pgr.reading.ac.uk

04 December 2023

Dear Editor,

Thank you for your letter advising a revision of our paper "**Stigma and its impact on disclosure and mental health secrecy in young people with depression symptomology: a qualitative analysis**".

Please see our comments to reviewers below and revised paper submitted. All amendments have been highlighted in yellow in our re-submitted paper.

Journal Requirements to Author:

When submitting your revision, we need you to address these additional requirements.

Please ensure that your manuscript meets PLOS ONE's style requirements, including those for file naming. The PLOS ONE style templates can be found at https://journals.plos.org/plosone/s/file?id=wjVg/PLOSOne_formatting_sample_main_body.pdf and https://journals.plos.org/plosone/s/file?id=ba62/PLOSOne_formatting_sample_title_authors_affiliations.pdf

- **Response.** Thank you very much for your positive feedback and thoughtful comments about our paper. We have incorporated PLOS ONE´s style requirements in our manuscript. We have removed any competing interest information in the declaration section.

Thank you for stating the following in the Funding Section of your manuscript:
"NW time on the project was funded by the European Research Council (ERC SOAR-851890). This project received no other funding."

We note that you have provided funding information that is not currently declared in your Funding Statement. However, funding information should not appear in the Acknowledgments section or other areas of your manuscript. We will only publish funding information present in the Funding Statement section of the online submission form.

Please remove any funding-related text from the manuscript and let us know how you would like to update your Funding Statement. Currently, your Funding Statement reads as follows:
"The author(s) received no specific funding for this work."

Please include your amended statements within your cover letter; we will change the online submission form on your behalf."

- **Response.** We have removed any funding-related text from the manuscript and would appreciate if you could update our Funding Statement as follow: “NW time on the project was funded by the European Research Council (ERC SOAR-851890). This project received no other funding.”

We note that you have indicated that data from this study are available upon request. PLOS only allows data to be available upon request if there are legal or ethical restrictions on sharing data publicly. For more information on unacceptable data access restrictions, please see http://journals.plos.org/plosone/s/data-availability#loc-unacceptable-data-access-restrictions.

In your revised cover letter, please address the following prompts:

a) If there are ethical or legal restrictions on sharing a de-identified data set, please explain them in detail (e.g., data contain potentially sensitive information, data are owned by a third-party organization, etc.) and who has imposed them (e.g., an ethics committee). Please also provide contact information for a data access committee, ethics committee, or other institutional body to which data requests may be sent.

b) If there are no restrictions, please upload the minimal anonymized data set necessary to replicate your study findings as either Supporting Information files or to a stable, public repository and provide us with the relevant URLs, DOIs, or accession numbers. For a list of acceptable repositories, please see http://journals.plos.org/plosone/s/data-availability#loc-recommended-repositories.

We will update your Data availability statement on your behalf to reflect the information you provide.

- **Response.** We have uploaded the minimal anonymized data set necessary to replicate our study findings through the University of Reading´s repository with the following reference:

  Prizeman, Katie (2023): Stigma and its impact on disclosure and mental health secrecy in young people with depression symptomology. University of Reading. Dataset. https://doi.org/10.17864/1947.000517

  We have also removed our data availability statement from our manuscript for the journal to update on our behalf.

Please include a separate caption for each figure in your manuscript.

- **Response.** We have included the following caption in our manuscript for our Figure 1: “Fig 1 shows a summary of participants' link research constructs in a visual form (thicker lines indicate a greater connection). This output was used to discuss how and why participants felt the constructs were connected.”

Reviewer's Responses to Questions:
Comments to the Author

1. Is the manuscript technically sound, and do the data support the conclusions?

The manuscript must describe a technically sound piece of scientific research with data that supports the conclusions. Experiments must have been conducted rigorously, with appropriate controls, replication, and sample sizes. The conclusions must be drawn appropriately based on the data presented.

Reviewer #1: Partly
Reviewer #2: Yes

---

2. Has the statistical analysis been performed appropriately and rigorously?

Reviewer #1: No
Reviewer #2: N/A

---

3. Have the authors made all data underlying the findings in their manuscript fully available?

The PLOS Data policy requires authors to make all data underlying the findings described in their manuscript fully available without restriction, with rare exception (please refer to the Data Availability Statement in the manuscript PDF file). The data should be provided as part of the manuscript or its supporting information, or deposited to a public repository. For example, in addition to summary statistics, the data points behind means, medians and variance measures should be available. If there are restrictions on publicly sharing data—e.g. participant privacy or use of data from a third party—those must be specified.

Reviewer #1: Yes
Reviewer #2: Yes

---

4. Is the manuscript presented in an intelligible fashion and written in standard English?

PLOS ONE does not copyedit accepted manuscripts, so the language in submitted articles must be clear, correct, and unambiguous. Any typographical or grammatical errors should be corrected at revision, so please note any specific errors here.

Reviewer #1: Yes
Reviewer #2: Yes

---

5. Review Comments to the Author:

Please use the space provided to explain your answers to the questions above. You may also include additional comments for the author, including concerns about dual publication, research ethics, or publication ethics. (Please upload your review as an attachment if it exceeds 20,000 characters)

Reviewer #1:

1. *The term youth was used in the title, and young people were used mostly throughout the manuscript. Author should consider either one of these terms (due to different age range these terms hold)*

- **Response.** Thank you very much for your positive feedback and thoughtful comments about our paper. This is a good point and we do agree with the reviewer and value the feedback regarding our term use. To address it we have changed the term `youth` to `young people` throughout the paper.

2. *In the Recruitment section, it is advised to explain how many subjects participated in the beginning, and how many were excluded due to low score MFQ.*

- **Response.** We have now included this information in Method section (p. 6).

3. *Either/not participants are taking medications would also affect their communication, mood and feelings.*

- **Response.** We take the reviewer's point regarding medication taking of our participants. That said, we did not collect data on this information. To address this, we have included a sentence in the Participants and Recruitment section: "Nor did we collect data on participant psychiatric medication." (p. 8)

4. *Despite of being a qualitative study, the MFQ score should be analyzed in relation to the themes (and sub-themes)*

- **Response.** We recruited only MFQ scores over 27, which is considered the clinical cut-off for high depression symptoms. Therefore, it is not clear to us how we would further delineate between depression symptoms in this sample. Further, as we only have 28 participants (due to qualitative data saturation), we have no statistical power to do a quantitative analysis between the MFQ data and the sub-themes.

  This being said, to address the reviewer's point, we have included the following information in the results section: "Participants' demographics and clinical characteristics are presented in Table 2. All participants had an MFQ of <27. A cut-off score of 27 and above has been identified as the difference between clinical and non-clinical levels of depressive symptoms (44)." (p. 12)

  We have also moved Table 2: Participants' demographics and clinical characteristics to the results section (p. 13).

Reviewer #2:

1. *Please revise as feedback, particularly in methods, result and discussion. In methods please add the major questionnaire, in result please make it connected to the research questions and in the discussion, please check citetation style.*

- **Response.** Thank you very much for your constructive feedback about our paper. We have included and referred to our major questionnaire in the methods section (Table 1) (p. 6-7).

- **Response.** We have included information in the results section to connect it to research questions. "Four interrelated broad themes emerged from the data. Themes were used to explore research questions 1–2, which aimed to better understand the subjective views of stigma among young people with depression symptoms and the influence it has on loneliness, social isolation, secrecy, and disclosure of depression." (p. 12).

  We have also included information throughout the result (p. 12-29) and discussion (p. 29-33) sections to better connect each theme back to our research questions.

- **Response.** We have amended any citation style mistakes throughout the paper.

---

6. PLOS authors have the option to publish the peer review history of their article (what does this mean?). If published, this will include your full peer review and any attached files.

If you choose "no", your identity will remain anonymous but your review may still be made public.

Do you want your identity to be public for this peer review? *For information about this choice, including consent withdrawal, please see our [Privacy Policy].*

Reviewer #1: No
Reviewer #2: No

Yours sincerely,

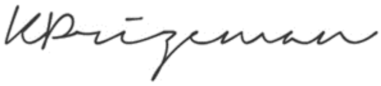

Katie Prizeman
